# Photocrosslinkable Trehalose Derivatives Carrying Mesogenic Groups: Synthesis, Characterization, and in Vitro Evaluation for Fibroblast Attachment

**DOI:** 10.3390/jfb7030024

**Published:** 2016-09-10

**Authors:** Shinya Yano, Naozumi Teramoto, Toshiaki Shimasaki, Mitsuhiro Shibata

**Affiliations:** Department of Applied Chemistry, Faculty of Engineering, Chiba Institute of Technology, 2-17-1 Tsudanuma, Narashino, Chiba 275-0016, Japan; shinya.y08232@gmail.com (S.Y.); shimasaki.toshiaki@it-chiba.ac.jp (T.S.); mitsuhiro.shibata@p.chibakoudai.jp (M.S.)

**Keywords:** trehalose, mesogen, liquid crystal, cinnamoyl, photocrosslinking, fibroblast, cell growth

## Abstract

A photocrosslinkable trehalose derivative carrying mesogenic groups was synthesized by esterification reactions. The derivative (TC-HBPHA) was synthesized by the reaction of partially cinnamoyl-modified trehalose (TC4) with 4-(4-hexyloxybenzoyloxy)phenoxy-6-oxohexanoic acid (HBPHA) as a mesogenic unit. TC-HBPHA showed a nematic liquid crystalline mesophase at a temperature range from 150 °C to 175 °C in the heating process under observation with a polarized optical microscope. The dimerization of the cinnamoyl groups of TC-HBPHA by ultraviolet (UV) light irradiation was monitored by ultraviolet-visible (UV-Vis) spectroscopy and Fourier transform infrared (FT-IR) spectroscopy. The photocrosslinked film was obtained after the UV irradiation of TC-HBPHA, and it kept the liquid crystalline mesophase at almost the same temperature range. Fibroblast cells cultured on the photocrosslinked TC-HBPHA proliferated as well as on the polystyrene culture plate, indicating that the film has no toxicity. Interestingly, some cells on photocrosslinked TC-HBPHA had a spindle shape and aligned characteristically.

## 1. Introduction

The liquid-crystal state is a unique phase with the characteristics of disordered liquid and ordered solid, and liquid crystal materials have been widely applied to products such as display devices and high performance polymer materials [1,2]. More recently, the photo-orientation technique of liquid crystal has been developing and attracting attention [3,4,5]. Besides display devices and high-performance materials, biomedical applications of liquid crystals are now in progress and opening up new research fields [6,7]. Indeed, as biological membranes consisting of phospholipids are known to be in a liquid-crystal state, the unfixed ordered state plays an important role in biological systems [6,8,9].

Crosslinkable liquid-crystal monomers and polymers are expected to expand applications of liquid crystals for use as nonlinear optical materials [10], tunable optical filters and polarizers [11,12], and stabilized liquid crystal systems [13]. Especially, photocrosslinkable liquid-crystal polymers can be used for the photo-orientation materials [14,15,16]. Though these materials are very attractive for use in biomedical materials, there are few studies reported [17,18,19,20]. Zhou and Yi [17] studied in vitro blood compatibility of composite films of polyurethane with three different liquid crystal molecules. They showed that the composite membranes of polyurethane and cholesteryl oleyl carbonate (COC) had hemocompatibility. The same research group (Li et al. [18]) also reported that polysiloxane containing 20 or 30 wt % of COC had good anti-coagulant properties. Shih et al. [19] also showed the hemocompatibility of COC-containing polyurethanes. Hwang et al. [20] reported the preparation of self-assembly of cholesterol-terminated oligo(l-lactic acid) and culturing of fibroblasts on their materials. They observed enhanced proliferation and adhesion on their self-assembling materials compared to poly(l-lactic acid). According to this research, materials derived from cholesterol, which is naturally occurring, are favorable for biocompatibility. Relating to toxicity of artificial liquid-crystal compounds, Woolverton et al. [21], Luk et al. [22], and Lockwood et al. [23] studied toxicity of liquid crystals to bacteria, mammalian cells, and human embryonic stem cells, respectively. Some liquid crystals are toxic and some are non-toxic.

**Scheme 1:**
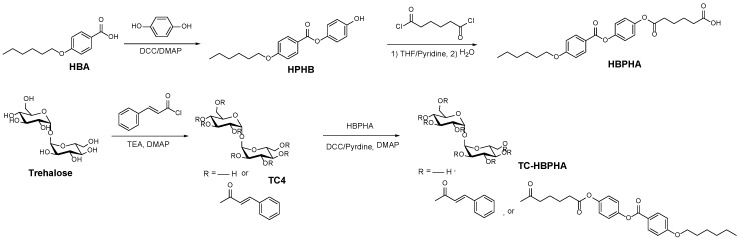
Synthesis of HBPHA, TC4, and TC-HBPHA.

Trehalose-derived materials are also receiving attention on the basis of their characteristic structure and biocompatibility [24,25,26,27]. John et al. [24] synthesized trehalose diesters by an enzymatic reaction using lipase. The trehalose diesters were found to be gelators for some organic solvents. Mancini et al. [25] synthesized glycopolymers carrying trehalose units at side chains by reversible addition-fragmentation chain transfer (RAFT) polymerization. They conjugated the glycopolymers to protein and found that the glycopolymers increased the stability of the protein. Burek et al. [26] synthesized diallyl compounds from trehalose and allyloxybenzaldehyde. They reported that the trehalose-based diallyl compounds can be used as a crosslinker of poly(*N*-isopropylacrylamide) hydrogels, and that the resulting hydrogels were non-toxic and degradable in acidic conditions. We also have developed trehalose-derived materials for a decade [28], and demonstrated fibroblast growth on photocrosslinked trehalose cinnamate (TC) [27]. Cinnamoyl groups are known to dimerize under ultraviolet (UV) light irradiation to yield a four-membered ring. This photo-dimerization reaction was often used for photocrosslinking of polymers [14,15,29,30,31,32]. Here we designed a trehalose-derived photocrosslinkable compound carrying cinnamoyl groups and mesogenic groups (Scheme 1). Liquid-crystal materials derived from saccharides are interesting materials, and their preparation and properties have been reported in the literature [33,34,35,36]. As recognized by Noller and Rockwell [33], higher alkylglycosides show liquid crystal behavior. Since then, many types of liquid crystalline glycoside derivatives and mesogenic sugars have been reported [34]. Among them, Kohne and Praefcke [35] reported the discotic liquid crystals from hexasubstituted inositols, and Tian et al. [36] reported a chiral nematic star-shaped (undecasubstituted) liquid crystal synthesized from melitose and mesogenic side arms. However, to our knowledge, there is no report on the biocompatibility of these types of materials.

In the present study, we synthesized photocrosslinkable trehalose derivatives carrying mesogenic groups by the esterification reaction (Scheme 1), and investigated their liquid crystal behaviour and biocompatibility. We intend to develop coating materials that can control cell alignment via the liquid crystalline photoalignment technique as a final goal. Cells such as fibroblasts which grow on scaffolds are known to have an effect via contact guidance, such as microscale topographies [37]. Very recently fibroblast cells cultured on oriented nanofibers were reported to upregulate genes associated with actin production, actin polymerization, and focal adhesion formation [38]. We expect that the materials we synthesized here will provide a first step for development of such materials that can control cell behavior.

## 2. Results and Discussion

### 2.1. Synthesis of a Trehalose Derivative Carrying Cinnamoyl Groups and Mesogenic Groups

**Figure 1:**
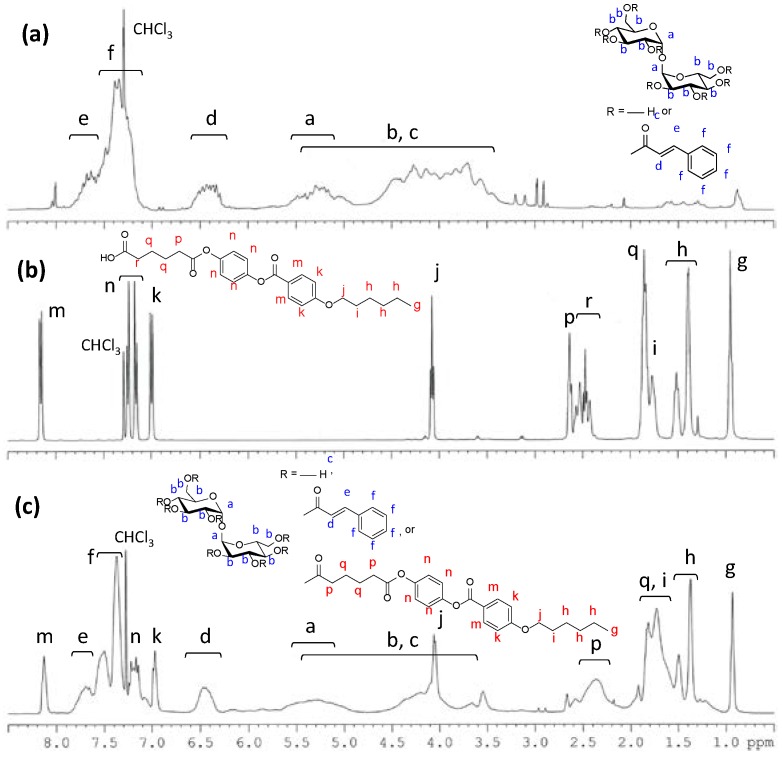
^1^H-NMR spectra of (**a**) TC4; (**b**) HBPHA; and (**c**) TC-HBPHA. CDCl_3_ was used as a solvent for the measurement.

HBPHA, a carboxylic compound with a mesogenic group, was synthesized using the method reported by Tian et al. [36]. We obtained HBPHA white powder after purification by silica gel column chromatography. The final step of the synthesis of a trehalose derivative carrying cinnamoyl groups and mesogenic groups (TC-HBPHA) was the reaction of partially cinnamoyl-modified trehalose with HBPHA. The reaction was carried out by the esterification reaction using *N*,*N*′-dicyclohexylcarbodiimide (DCC) as a condensation reagent. The reaction needed a long time (72 h) because of hindrance of the reaction site. Furthermore, the crude product included a significant amount of impurities such as unreacted DCC, unreacted HBPHA, and *N*,*N*′-dicyclohexylurea. Therefore we needed to purify the product by washing many times with various solvents. The yield became low after the purification steps. Figure 1 shows the proton nuclear magnetic resonance (^1^H-NMR) spectra of TC4, HBPHA and TC-HBPHA. Each signal in the ^1^H-NMR spectrum of HBPHA (Figure 1b) can be assigned to each proton on the chemical structure except for the signal at 1.6 ppm, which is assigned to a trace of water. As reported in the previous literature [39], peaks observed in the spectrum of TC4 (Figure 1a) were broadened, implying that the product contains some variation of trehalose derivatives modified with a varied number of cinnamoyl and mesogenic groups, i.e., with various degrees of substitution (DS). Here, DS represents the number of the substituents based on one trehalose unit. Peaks observed in TC-HBPHA were also broadened, because TC-HBPHA was synthesized from TC4. In Figure 1c, proton signals assigned to aromatic and vinylene groups were observed at 8.2–6.3 ppm. Methine signals of the trehalose unit of TC-HBPHA were significantly broadened and observed at 5.7–3.4 ppm. Methylene and methyl signals of hexyloxy and 6-oxohexanoyl groups were observed at 4.0 and 2.5–0.9 ppm. The average DS for cinnamoyl groups of TC-HBPHA was 3.0, and the average DS for mesogenic groups of TC-HBPHA was 2.1, when calculated from the elemental analysis data. TC-HBPHA was soluble in many organic solvents such as *N*,*N*-dimethylformamide (DMF), dimethyl sulfoxide (DMSO), tetrahydrofuran (THF), acetone, chloroform, diethyl ether, and toluene. It was insoluble in water, methanol, ethanol, and hexane.

### 2.2. Photocrosslinking of TC-HBPHA

**Scheme 2:**
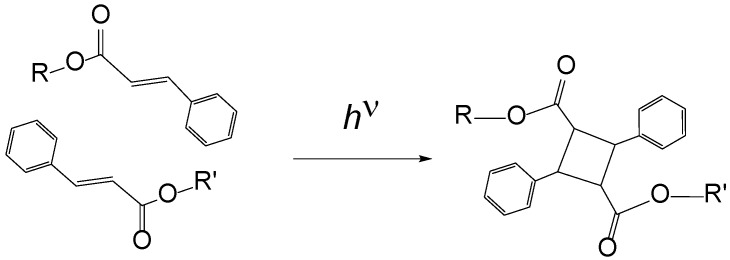
Dimerization of cinnamoyl groups by UV irradiation.

**Figure 2:**
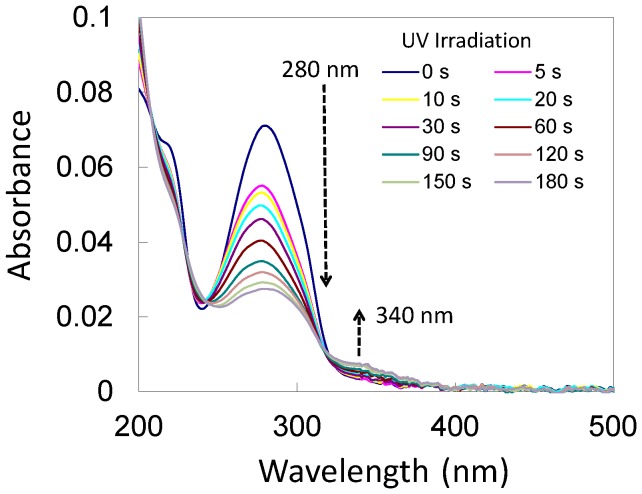
UV-Vis spectral change of a TC-HBPHA thin film by UV irradiation.

**Figure 3:**
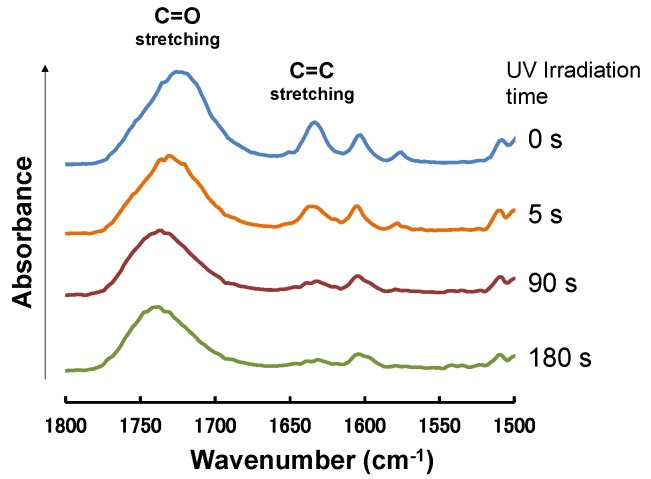
FT-IR spectral change of a TC-HBPHA thin film by UV irradiation.

Cinnamoyl groups are known to undergo dimerization by irradiation of UV light (Scheme 2). Because TC-HBPHA has several cinnamoyl groups in one molecule, photocrosslinking of TC-HBPHA occurs by UV irradiation. Generally, the dimerization reaction is monitored by the UV-Vis spectral analysis [40]. Figure 2 shows the UV-Vis spectral change during UV irradiation. During the course of UV irradiation, the absorbance at 280 nm decreased significantly. On the other hand, the absorbance at 340 nm slightly increased simultaneously. These results imply that the isomerization of cinnamoyl groups from the trans-form to the cis-form occurred to some extent during the UV irradiation, though dimerization of cinnamoyl groups predominated [41]. The isomerization of cinnamoyl groups was not observed at UV irradiation of TC4 in our previous report [39]. We considered that the isomerization observed here occurred at some cinnamoyl groups hindered by mesogenic groups. The reaction was also monitored by Fourier transform infrared (FT-IR) spectroscopy [42,43]. Figure 3 shows the FT-IR spectral change of TC-HBPHA during UV irradiation. The absorbance at 1722 cm^−1^ in the spectrum of non-irradiated TC-HBPHA corresponds to the cinnamoyl C=O stretching vibration conjugated with the adjacent C=C bond. This peak seems broadened because it contains the absorption of ester C=O vibrations of the mesogenic groups. The absorbance of cinnamoyl C=O vibration is known to decrease and shift to the higher wavenumber region by the dimerization reaction because of deconjugation [39,42]. The absorbances at 1632 cm^−1^ and 1574 cm^−1^ correspond to the C=C symmetric and asymmetric stretching vibration, respectively. These absorbances significantly decreased, which suggests that dimerization reaction occurred under UV irradiation [43].

### 2.3. Liquid Crystalline Behavior of TC-HBPHA before and after UV Irradiation

**Figure 4:**
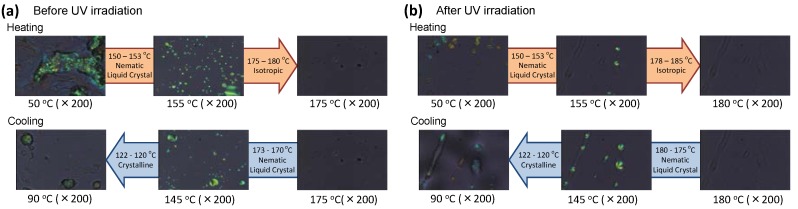
Polarized optical micrographs under crossed Nicols of TC-HBPHA (**a**) before UV irradiation and (**b**) after UV irradiation.

The liquid crystalline behavior of TC-HBPHA was observed using a polarized optical microscope (POM). Before UV irradiation, TC-HBPHA showed a droplet texture and the morphology of nematic liquid crystal (Figure 4a). The droplet texture was also reported for the sugar-derived liquid crystal [36]. The liquid crystalline morphology was observed from 150 °C to 175 °C at the heating process, and observed from 170 °C to 120 °C at the cooling process. The liquid crystalline droplets moved around in these temperature regions. The fact that the liquid crystalline droplets did not fill the space implies the partial existence of liquid crystal regions in the isotropic non-liquid crystalline matrix. We considered that this inhomogeneity was caused by the variation of DS of TC-HBPHA for mesogenic groups: liquid crystalline regions may consist of TC-HBPHA with higher DS, and non-liquid crystalline regions may consist of TC-HBPHA with lower DS.

Figure 4b shows the liquid crystalline behavior of TC-HBPHA after UV irradiation. Interestingly, though the droplet texture was also observed, the mobility of the droplets was limited. The liquid crystalline morphology of irradiated TC-HBPHA was observed from 150 °C to 180 °C at the heating process, and observed from 175 °C to 120 °C at the cooling process. Many reactive mesogens were investigated for liquid crystalline thermosets; some are known to lose liquid crystalline properties and keep mesomorphic structure in the solid phase, and some are known to keep the partial liquid crystalline properties [44]. The photocrosslinked TC-HBPHA corresponds to the latter case. We found that the photocrosslinking mainly occurred in the non-liquid crystalline matrix described above, and that mesomorphic properties of the liquid crystalline regions were kept.

**Figure 5:**
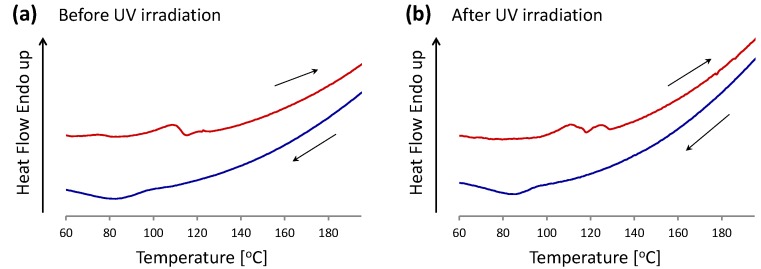
DSC thermograms of TC-HBPHA (**a**) before and (**b**) after UV irradiation.

We measured thermal properties of TC-HBPHA by DSC before and after UV irradiation intending to clarify the phase transition behavior. Figure 5 shows the differential scanning calorimetry (DSC) thermograms of TC-HBPHA before and after UV irradiation. However, we could not determine the apparent transition points corresponding to the results observed using a POM. In particular, we could not find the transition point between the nematic phase and the isotropic phase in the DSC analysis. Furthermore the solid-nematic phase transition was observed as broadened and complex peaks in the DSC analysis after UV irradiation as well as before UV irradiation. However, several endothermic peaks were observed at a lower temperature region than expected from the observation with a POM. As described above, we can see the partial liquid crystalline behavior, possibly depending on the number of mesogenic groups in a TC-HBPHA molecule. Since the product contains TC-HBPHA molecules carrying various numbers of mesogenic groups, it is reasonable that the transition point is not obvious.

### 2.4. Cell Culture on UV-Irradiated TC-HBPHA and Study of Surface Hydrophobicity

**Figure 6:**
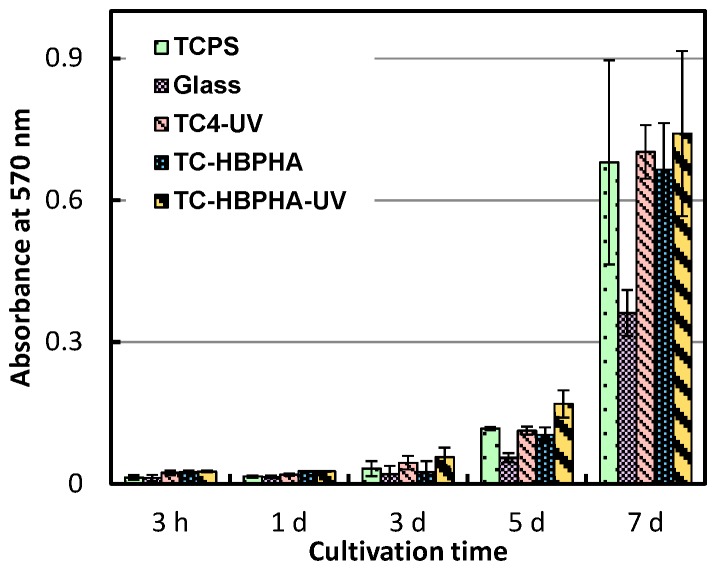
Cell growth evaluated by MTT test on TCPS, glass cover slips, UV-irradiated TC4, non-irradiated TC-HBPHA, and UV-irradiated TC-HBPHA.

**Figure 7:**
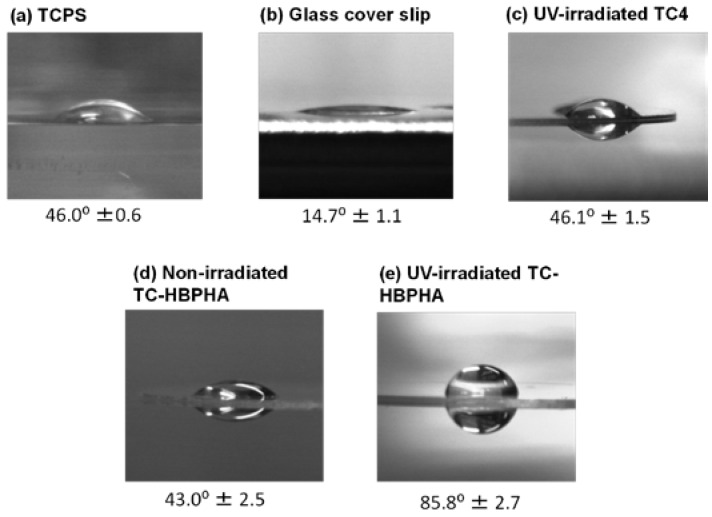
Contact angles and photographs of a water drop placed on (**a**) TCPS; (**b**) a glass cover slip; (**c**) UV irradiated TC4; (**d**) non-irradiated TC-HBPHA; and (**e**) UV-irradiated TC-HBPHA.

**Figure 8:**
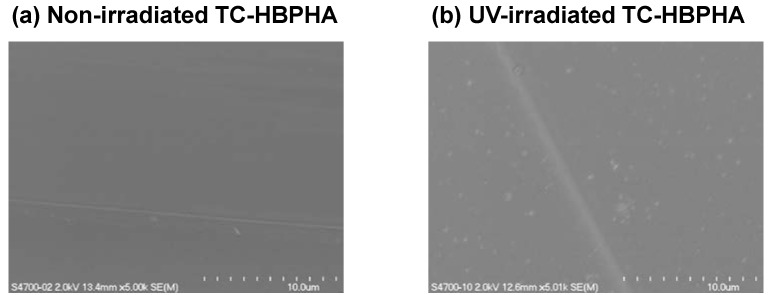
Surface morphology of (**a**) non-irradiated TC-HBPHA and (**b**) UV-irradiated TC-HBPHA observed by FE-SEM.

**Figure 9:**
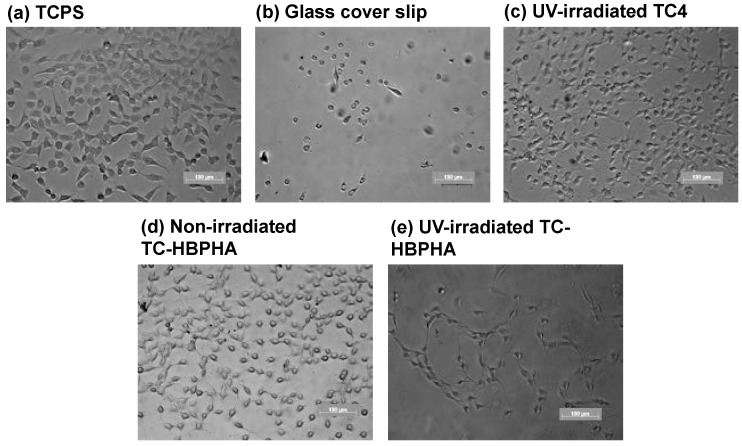
Morphology of cells grown on (**a**) TCPS; (**b**) a glass cover slip; (**c**) UV irradiated TC4; (**d**) non-irradiated TC-HBPHA; and (**e**) UV-irradiated TC-HBPHA after 5-d culture observed by phase-contrast microscope.

Cell viability and growth on the UV-irradiated and non-irradiated TC-HBPHA thin films were tested using 3T3 Swiss Albino fibroblast cells. The cell observation after tripan-blue staining revealed that there are very few dead cells on all of the tested samples. Figure 6 shows the result of cell growth evaluation using the MTT test. The result revealed that the fibroblast cells increased both on the UV-irradiated and non-irradiated TC-HBPHA as well as tissue culture polystyrene (TCPS) and UV-irradiated TC4. Generally, cell proliferation is influenced largely by the surface hydrophobicity of the substrate. We studied surface hydrophobicity of our samples using a contact angle meter with a water drop on substrates. Figure 7 shows the photograph and the contact angle of a water drop on each substrate. As a result, UV-irradiated TC-HBPHA had especially higher hydrophobicity, despite its good cell compatibility. The contact angle is known to be influenced by the surface morphology, and the surface of UV-irradiated TC-HBPHA was observed using FE-SEM. The SEM images are shown in Figure 8. Many small spots were observed on the surface of UV-irradiated TC-HBPHA, while the surface of non-irradiated TC-HBPHA was almost homogeneous. This result suggests that some rearrangement of mesogenic groups and phase separation possibly occurred during UV irradiation [45], causing the increase of hydrophobicity. We carefully observed the cell morphology on each substrate after 5-d culture, and found that many cells adhering on UV-irradiated TC-HBPHA were characteristically aligned and had a spindle shape (Figure 9). This phenomenon usually occurs on the physically patterned substrate [46,47]. Though we do not have a clear vision of this result at the current stage, we considered two possible reasons. First, the cells can be considered to attach on the substrate, avoiding certain regions. We observed the separated liquid crystalline regions by the POM observation and their fixation by UV irradiation as described above. The cells may have avoided this separated liquid crystalline phase. Second, the cells can be considered to favorably attach some regions. We reported the favorable cell adhesion on the UV-irradiated TC in the previous paper [27]. As described above, some regions are considered to contain relatively more cinnamoyl groups because of variation of the DS. In either case, the cell behavior is considered to be due to the phase separation of liquid crystalline regions.

## 3. Materials and Methods

### 3.1. Materials

Trehalose dihydrate was kindly provided by Hayashibara Co., Ltd. (Okayama, Japan) and dehydrated at 130 °C for 24 h before use. Cinnamoyl chloride, 4-(dimethylamino)pyridine (DMAP), 4-hexyloxybenzoic acid, adipoyl chloride were purchased from Tokyo Kasei Kogyo Co. (Tokyo, Japan). Anhydrous *N*,*N*-dimethylformamide (DMF) and *N*,*N*′-dicyclohexylcarbodiimide (DCC) were purchased from Sigma-Aldrich Co. (St Louis, MO, USA). Triethylamine, hydroquinone, sodium bicarbonate, sodium sulfate, oxalic acid and other organic solvents were purchased from Kanto Chemical Co. (Tokyo, Japan). These reagents and solvents were used as received.

### 3.2. Synthesis of Partially Cinnamoyl-Modified Trehalose (TC4)

TC4 was prepared by the method reported previously [39]. Dehydrated trehalose (10 mmol) was finely suspended in anhydrous DMF (15 mL) at 60 °C, and triethylamine (TEA) (40 mmol) and 4-(dimethylamino)pyridine (DMAP) (5 mmol) were added to the suspension. A solution of cinnamoyl chloride (40 mmol) in anhydrous DMF (5 mL) was added to the mixture dropwise. After stirring at room temperature for 24 h, the reaction product was obtained by precipitation in deionized water and washed twice with deionized water. White powder was obtained after drying in vacuo for 2 days (yield, 55%).

### 3.3. Synthesis of 4-Hydroxyphenyl 4'-Hexyloxybenzoate (HPHB)

4-(Hexyloxy)benzoic acid (HBA) (15 mmol) was dissolved in an anhydrous dichloromethane (30 mL)/DMF (20 mL) mixed solvent at room temperature, and DMAP (1.5 mmol) and hydroquinone (60 mmol) were added to the solution. A solution of *N*,*N*′-dicyclohexylcarbodiimide (DCC) (22.5 mmol) in an anhydrous dichloromethane (15 mL)/DMF (10 mL) mixed solvent was added. After stirring at 30 °C for 24 h, the reaction mixture was filtered and the filtrate was concentrated in vacuo. To the concentrate was added excess amount of dichloromethane, followed by washing with a saturated sodium bicarbonate aqueous solution. After washing with deionized water, the organic layer was dried with sodium sulfate overnight. The crude product was obtained by the evaporation of the solvent, and further purified by silica gel column chromatography (eluent: dichloromethane). White powder was obtained after drying in vacuo for 24 h (yield, 58%).

### 3.4. Synthesis of 6-(4-Hexyloxybenzoyloxy)phenoxy-6-oxohexanoic Acid (HBPHA)

HBPHA was prepared by the method reported by Tian et al. [36]. HPHB (18 mmol) was dissolved in an anhydrous tetrahydrofuran (THF) (16 mL)/pyridine (2 mL) mixed solvent at room temperature. A solution of adipoyl chloride (36 mmol) in anhydrous THF (9 mL) was added to the mixture dropwise. After stirring at room temperature for 15 h, the reaction product was obtained by precipitation in deionized water and washed twice with deionized water. After drying in vacuo for 24 h, the product was purified by silica gel column chromatography (eluent: dichloromethane:acetone = 30:1). White powder was obtained after drying in vacuo for 24 h (yield, 47%).

### 3.5. Synthesis of a Trehalose Derivative Esterified with Cinnamoyl Groups and HBPHA (TC-HBPHA)

TC4 (0.97 mmol) and HBPHA (4.86 mmol) was dissolved in anhydrous pyridine (11 mL) at room temperature, and DMAP (2.92 mmol) was added to the solution. After a solution of DCC (5.83 mmol) in pyridine (30 mL) was added, the mixture was stirred at room temperature for 72 h. The reaction product was obtained by filtering the reaction mixture and concentrating the filtrate in vacuo. The crude product was obtained by precipitation in deionized water and washed twice with deionized water. Then the crude product was dissolved in dichloromethane and dried with sodium sulfate overnight. After evaporation of the solvent, the product was washed with ethanol. Furthermore the product was washed with an oxalic acid (5.84 mmol) solution in methanol (10 mL) to deactivate DCC. The product was redissolved in dichloromethane and washed with a saturated sodium bicarbonate aqueous solution. After washing with deionized water, the solution was dried with sodium sulfate overnight and evaporated to dryness. The resulting solid product was dissolved in a small amount of chloroform and reprecipitated in hexane. White powder was obtained after drying in vacuo for 24 h (yield, 8%).

TC-HBPHA: IR (KBr, cm^−1^): 2933, 2852 (CH, CH2, CH3), 1724 (C=O), 1633 (C=C), 1602 (aromatic C–C), 1575 (C=C), 1510, 1496, 1450 (aromatic C–C), 1255, 1165 (C–O–C).

^1^H-NMR (CDCl_3_, TMS, δ, ppm): 8.13 (d, *J* = 7.6 Hz, *H*-Ar (benzoyloxy)), 7.82–7.61 (m, *H*–C=C), 7.60–7.45 (m, *H*-Ar (cinnamoyl)), 7.45–7.30 (s, *H*-Ar (cinnamoyl)), 7.26–7.01 (m, *H*-Ar (phenoxy)), 6.98 (d, *J* = 7.5 Hz, *H*-Ar (benzoyloxy)), 6.60–6.33 (m, *H*–C=C–), 5.7–4.9 (m, *H*-trehalose unit), 4.7–3.8 (m, *H*-trehalose unit, –C*H*_2_–O– (hexyloxy)), 2.5–2.3 (m, –C*H*_2_–C(=O)– (oxohexanoyl)), 1.9–1.5 (m, –O–CH_2_–C*H*_2_– (hexyloxy), –C*H*_2_–CH_2_–C(=O)– (oxohexanoyl)), 1.51 (s, –C*H*_2_– (hexyl)), 1.39 (s, –C*H*_2_– (hexyl)), 1.29 (s, –C*H*_2_– (hexyl)), 0.95 (s, –C*H*_3_ (hexyl)).

Anal. Found: C, 67.54; H, 6.21; O, 26.09.

### 3.6. Photocrosslinking of a Trehalose Derivative Carrying Mesogenic Groups

For preparation of films used in the ultraviolet-visible (UV-Vis) spectroscopy, quartz glass plates were coated with samples using an Aiden DC4100 dip coater (Aiden Co. Ltd., Kobe, Japan) as reported previously [27]. Chloroform solutions of samples at a concentration of 10 mg/mL were used. The dipping time was 1 min and the speed of subsequent raising was 0.5 mm/s. Prior to the dipcoating process, glass substrates were washed with the mixed acid solution of concentrated nitric acid, concentrated sulfuric acid, and water (1:3:6). In order to crosslink at the liquid crystalline state, samples were once heated at 180 °C and cooled to 145 °C. Then, the samples were irradiated at 145 °C using an Ushio SP-7 spot UV irradiator (Ushio Inc., Tokyo, Japan) with a 250-W deep UV lamp through a λ ≥ 280 nm long pass filter (Thorlabs Japan Co., Ltd., Tokyo, Japan) to cut the shorter-wavelength light. The distance between the sample and the light guide was 17 cm, and the light intensity was 60 mW/cm^2^.

### 3.7. Characterization

Proton nuclear magnetic resonance (^1^H-NMR) spectra were recorded on a Bruker AV-400 spectrometer (Bruker Corp., Karlsruhe, Germany) using CDCl_3_ as a solvent. Elemental analysis was carried out for C, H, N and O using an Exeter Analytical CE-440 elemental analyser (Exeter Analytical Inc., Chelmsford, MA, USA). The average number of cinnamoyl groups and mesogenic groups substituted for hydroxyl groups on one trehalose unit was defined as the degree of substitute (DS) and calculated from the elemental analysis data. We found no nitrogen in the final product, TC-HBPHA. UV-Vis spectra were recorded on a JASCO V-650 spectrophotometer (JASCO Corp., Tokyo, Japan) with an attachment for the measurement of plates and films. Fourier transform infrared (FT-IR) spectra were recorded on a Shimadzu FT-IR 8400S spectrometer (Shimadzu Corp., Kyoto, Japan) by the KBr-pellet method. Differential scanning calorimetry (DSC) thermal curves were recorded on a PerkinElmer Pyris 1 differential scanning calorimeter (PerkinElmer Inc., Waltham, MI, USA). The heating rate and the cooling rate were 10 °C/min. Liquid crystal morphologies of samples on a glass slip was observed by an Olympus BS50 polarized optical microscope (POM) and an Olympus TH3 light source (Olympus Corp., Tokyo, Japan) equipped with a thermal stage. The surface morphologies of photo-cured samples were observed by a Hitachi S-4700 field emission scanning electron microscope (FE-SEM) (Hitachi High-Technologies Corp., Tokyo, Japan). The accelerating voltage was 1 kV, and the samples were coated with gold prior to the observation. The water contact angle was measured by an Excimer SImage mini contact angle meter (Excimer Inc., Yokohama, Japan). A 4-μL droplet of ultrapure water was placed at five positions on each sample followed by taking a digital picture to calculate static contact angles, and the five values were averaged.

### 3.8. Cell Culture Study

Round-shape glass cover slips (15 mm diameter) were coated with samples by the dipcoating method, and irradiated with UV light by an Ushio SP-7 spot UV irradiator. The irradiated cover slips were placed in a 24-well polystyrene (PS) culture plate and sterilized with ethylene oxide gas. After washing with D-MEM, 3T3 Swiss Albino mouse embryo fibroblast cells were seeded on the samples at 3 × 10^3^ cells/mL. The cells were incubated at 37 °C in 300 μL of D-MEM containing 10% FBS and 1% penicillin–streptomycin using a CO_2_ incubator with 5% CO_2_. Cell growth was observed using a Carl-Zeiss Axio Vert.A1 phase-contrast microscope (Carl Zeiss AG, Oberkochen, Germany). The number of cells adhered onto each sample was determined using a hemocytometer after treated with 0.25% trypsin–EDTA solution and trypan blue staining. Cell proliferation was also measured by MTT assay, which is based on the mitochondrial activity of viable cells. An MTT solution (300 μL of 1.0 mg/mL) was added to the samples and incubated for 90 min at 37 °C under 5% CO_2_. A 300 μL of a cell lysing solution containing 10% polyoxyethylene octylphenyl ether (NP-40) was added to the solution. The absorbance of the resulting solution at 570 nm was measured using a Bio-Rad iMark microplate reader (Bio-Rad Laboratories Inc., Hercules, CA, USA) (*n* = 4 for each assay).

## 4. Conclusions

Photocrosslinkable trehalose derivative carrying cinnamoyl groups and mesogenic groups were synthesized mainly by esterification reactions. From the UV-Vis and FT-IR spectral measurement of the product, the cinnamoyl groups mainly underwent dimerization, and some part underwent trans-cis isomerization by UV irradiation. We can observe a droplet morphology of the product under crossed Nicols from 150 °C to 175 °C in the heating process and from 170 °C to 120 °C in the cooling process before UV irradiation. After UV irradiation, a similar droplet morphology with limited mobility was observed from 150 °C to 180 °C at the heating process and from 175 °C to 120 °C at the cooling process. However, the transition points were not observed clearly in the DSC measurement. The fibroblast cell culture on TC-HBPHA thin films cast on glass cover slips revealed that TC-HBPHA did not have toxicity and has good cell compatibility before and after UV irradiation as well as a polystyrene culture plate. Many cells on UV-irradiated TC-HBPHA presented the characteristic alignment and a spindle shape. Though the reason for this alignment and shape is not clear at the current stage, it is interesting that the cell behavior is influenced by the substrate containing mesogenic groups. This is the first step to investigate cell behavior on photocrosslinked substrates containing mesogenic groups with liquid crystalline properties. Future studies will be conducted on cell behavior on the molecular-aligned substrate using liquid crystal materials.
